# Bimodal reflectance and fluorescence multispectral endoscopy based on spectrally resolving detector arrays

**DOI:** 10.1117/1.JBO.24.3.031009

**Published:** 2018-10-24

**Authors:** A. Siri Luthman, Dale J. Waterhouse, Laura Ansel-Bollepalli, Jonghee Yoon, George S. D. Gordon, James Joseph, Massimiliano di Pietro, Wladyslaw Januszewicz, Sarah E. Bohndiek

**Affiliations:** a University of Cambridge, Department of Physics, Cambridge, United Kingdom; b University of Cambridge, Cancer Research UK Cambridge Institute, Li Ka Shing Center, Robinson Way, Cambridge, United Kingdom; c University of Cambridge, Department of Engineering, Cambridge, United Kingdom; d University of Cambridge, MRC Cancer Unit, Hutchison/MRC Research Centre, Cambridge, United Kingdom

**Keywords:** spectrally resolving detector arrays, endoscopy, multiplexed, multispectral, hyperspectral, fluorescence, biomedical

## Abstract

Emerging clinical interest in combining standard white light endoscopy with targeted near-infrared (NIR) fluorescent contrast agents for improved early cancer detection has created demand for multimodal imaging endoscopes. We used two spectrally resolving detector arrays (SRDAs) to realize a bimodal endoscope capable of simultaneous reflectance-based imaging in the visible spectral region and multiplexed fluorescence-based imaging in the NIR. The visible SRDA was composed of 16 spectral bands, with peak wavelengths in the range of 463 to 648 nm and full-width at half-maximum (FWHM) between 9 and 26 nm. The NIR SRDA was composed of 25 spectral bands, with peak wavelengths in the range 659 to 891 nm and FWHM 7 to 15 nm. The spectral endoscope design was based on a “babyscope” model using a commercially available imaging fiber bundle. We developed a spectral transmission model to select optical components and provide reference endmembers for linear spectral unmixing of the recorded image data. The technical characterization of the spectral endoscope is presented, including evaluation of the angular field-of-view, barrel distortion, spatial resolution and spectral fidelity, which showed encouraging performance. An agarose phantom containing oxygenated and deoxygenated blood with three fluorescent dyes was then imaged. After spectral unmixing, the different chemical components of the phantom could be successfully identified via majority decision with high signal-to-background ratio (>3). Imaging performance was further assessed in an *ex vivo* porcine esophagus model. Our preliminary imaging results demonstrate the capability to simultaneously resolve multiple biological components using a compact spectral endoscopy system.

## 1 Introduction

Endoscopy is a clinical procedure that is used to visualize the epithelial surfaces of hollow organs. A common application of endoscopy is diagnosis and monitoring of abnormalities in the gastrointestinal (GI) tract. Early detection of GI cancers is an important application of endoscopy, given the potential to improve the prognosis.^1^ Several programs of population screening^2^ and high-risk patient surveillance^3^ exist to detect early signs of cancer in the GI tract based on white light endoscopy (WLE). During WLE, broadband white light in the visible spectral region illuminates the tissue and images are acquired in reflectance mode. WLE detects cancerous lesions based on structural changes or discoloration of the epithelial surface^1^ and may be used to guide the acquisition of tissue biopsies.^4^ Unfortunately, precancerous lesions, such as dysplasia, are often flat and do not exhibit detectable discoloration relative to normal tissue in the normal three reflectance color channels (RGB) used in WLE.^1^^,^^4^ This makes disease detection and acquisition of targeted biopsies with conventional WLE challenging.^1^^,^^4^ Consequently, there is a strong clinical interest in the development of new endoscopic screening methods that may enhance the contrast for precancerous lesions, enabling better targeting of biopsies and improving diagnostic performance.^1^^,^^4^^–^^10^

Multimodal endoscopy holds potential to improve the diagnostic performance of endoscopic cancer screening^11^ by simultaneously interrogating numerous contrast mechanisms linked to cancer. Combining reflectance-based imaging in the visible wavelength range with exogenous targeted fluorescent contrast agents in the near-infrared (NIR) wavelength range has recently attracted particular attention.^12^^,^^13^ Reflectance-based imaging yields image contrast via the absorption and scattering of visible light; these data could be used to determine clinical parameters, such as the oxygenation status of blood.^11^ Using targeted fluorescent contrast agents is advantageous as it can enhance molecular specificity for the disease type of interest, and when applied in the NIR, provides a relatively high signal-to-background ratio (SBR).^1^^,^^5^^,^^10^ Combining the two techniques enables both functional imaging, via reflectance imaging, and molecular imaging, with the targeted fluorescent contrast agents.

Many clinical endoscopes already acquire spectral data; however, the added value of methods such as narrowband imaging (NBI; two illumination bands and RGB detection), autofluorescence imaging (AFI; two illumination bands and monochrome detection), or trimodal imaging (combination of NBI and AFI with high definition WLE) for improving the detection rates of early dysplastic lesions remains unclear.^6^ The limited spectral range of these methods only provides a qualitative visualization of the epithelium. Methods with a finer sampling of the spectral response, such as multi- or hyperspectral imaging (MSI/HSI) systems (≈10 and 100 spectral color channels, respectively),^12^^,^^13^ may enable quantitative assessment of functional tissue properties and more detailed statistical classification of the spectral changes that occur during disease. The use of reflectance-based MSI/HSI systems in biomedical applications has, for example, been shown to increase contrast in vascular conditions,^14^ wound healing,^15^^–^^17^ ophthalmology,^15^^,^^18^^,^^19^ cancer diagnostics,^12^^,^^20^^–^^23^ and for the determination of tumor resection margins.^24^

The application of a targeted fluorescent contrast agent is often referred to as optical molecular imaging (OMI) and has shown promise for endoscopic cancer detection in the GI tract.^1^^,^^5^^,^^10^ An OMI contrast agent consists of a fluorescent dye conjugated to a targeting moiety, designed to highlight a biological process that is dysregulated in the disease state of interest.^1^^,^^5^ This may yield early diagnosis as the biological response (e.g., upregulation of a particular cell surface receptor) that may precede visible structural changes.^1^^,^^5^ Many OMI contrast agents are under preclinical^1^^,^^25^ and clinical investigation.^1^^,^^6^^,^^26^ In clinical endoscopic studies, OMI has been used to provide a “red flag,” prompting tissue biopsy in studies of colorectal^27^ and esophageal cancer.^6^ These early studies have indicated that endoscopic OMI may reduce the number of required biopsies and improve the early detection rates of dysplastic lesions.^6^^,^^27^ Typically, these studies have employed a dedicated single-band fluorescence channel, where an imaging fiber bundle is introduced into the accessory channel of a standard clinical endoscope (the so-called “babyscope” method) to relay the fluorescence information to a dedicated NIR camera system.^6^^,^^27^ As a result, usually only a single OMI contrast agent can be visualized, preventing multiplexed assessment of several biological pathways,^1^^,^^5^ or corrections for contrast agent binding nonuniformities,^1^^,^^5^^,^^10^^,^^28^ which would improve robustness to interpatient variability in screening.^5^

Acquiring spatiospectral data in both the visible and NIR wavelength regions presents an opportunity to perform reflectance-based imaging and OMI simultaneously. Previously reported flexible MSI or HSI endoscopes operate on the same babyscope model used in many OMI studies. Spectral imaging is typically achieved by filtering the light on either the illumination or detection side. Filtering has been achieved by the use of: a set of dichroic beamsplitters and dedicated detectors;^10^^,^^27^^,^^29^ a filter wheel with a set of bandpass filters;^21^^,^^30^^,^^31^ tunable filters;^23^^,^^32^ or a monochromator.^33^ Alternatively, a set of laser lines may be used for illumination.^7^^,^^29^^,^^34^^,^^35^ These systems either sacrifice temporal resolution for spectral data acquisition or require multiple dedicated detectors and spectral filters,^7^^,^^36^ making them bulky and expensive. There are a few alternative approaches to spectral endoscopy;^37^^–^^39^ however, to the best of our knowledge, these systems have yet to be demonstrated in clinical applications.

Here, we created a multispectral endoscope based on snapshot spectrally resolving detector arrays (SRDAs)^40^^,^^41^ to explore their ability to deliver a spectral endoscopy system with potential for a high speed and low cost to enable multiplexed reflectance-based imaging and OMI. Two SRDAs were integrated into a bimodal endoscope to allow for simultaneous reflectance-based imaging in the visible wavelength range and multiplexed fluorescence imaging in the NIR using combined excitation from a broadband white light source and dedicated NIR laser. We present the design and technical characterization of the prototype multispectral endoscope, including a computational model that was used to predict and optimize the component-dependent endoscopic imaging performance prior to system assembly. We also demonstrate simultaneous imaging and spectral unmixing of chemically oxygenated and deoxygenated mouse blood with three fluorescent dyes in an agarose phantom, as well as imaging in an *ex vivo* porcine model for further endoscopic performance evaluation. This paper shows the first steps toward clinical translation of SRDA-based endoscopy.

## 2 Methods

### 2.1 Endoscope Design and Assembly

**Fig. 1:**
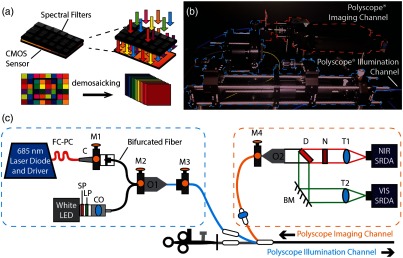
Optical setup of the bimodal endoscope to achieve simultaneous spectral reflectance imaging in the visible and multiplexed fluorescence imaging in the NIR spectral range. (a) The spectrally resolving detector arrays (SRDAs, simplified schematic) consist of spectral filters deposited on CMOS sensors in a mosaic pattern (bottom left) forming spectral macropixels (denoted in the black square). The MSI data can be extracted postdata acquisition, by interpolating between pixels in adjacent spectral macro-pixels (bottom right). (b) Photograph of the experimental system. (c) Optical layout of the endoscope, which was assembled around a CE marked commercial modular endoscope (PolyScope^®^). Color annotations illustrate the illumination and imaging channels in panels (b) and (c). Key: NIR SRDA, near-infrared spectrally resolving detector array; VIS SRDA, visible spectrally resolving detector array; T1, tube lens for NIR SRDA; N, 685-nm notch filter; D, 650-nm dichroic beam splitter; T2, tube lens for visible SRDA; BM, broadband mirror; O2, 20× objective lens; O1, 40× objective lens; CO, LED to fiber coupler; LP, 465-nm long-pass filter; SP, 650-nm short pass filter; C, FC/PC adjustable collimator; FC/PC, single-mode patch cable; M, x-y-z adjustable mounts (M1, collimator to bifurcated fiber; M2, bifurcated fiber to O1; M3, endoscope illumination channel adapter; M4, endoscope imaging channel to O2).

A bimodal endoscope incorporating the visible and NIR SRDAs [Fig. 1(a)] was assembled to allow simultaneous visible reflectance-based imaging and NIR fluorescence imaging based on illumination from a white light broadband LED and an NIR laser diode [Fig. 1(b)]. The endoscope was assembled around a CE marked commercial babyscope (PolyScope^®^; PolyDiagnost), which may be used independently or introduced through the accessory channel of another endoscope,^42^ to facilitate direct future translation to clinical endoscopic imaging applications. The PolyScope consists of a flexible optical system (PD-PS-0095, PolyScope; PolyDiagnost) and a disposable catheter, with a maximum diameter of 3 mm, through which the optical system is threaded to protect it from patient contact during imaging. The flexible optical system consists of a fiber bundle with 10,000 pixels of 500-μm diameter for imaging and a polymethyl methacrylate light guide with numerical aperture of 0.63 for illumination. In addition to the flexible optical system, the PolyScope also contains a 1.2-mm working channel, not used in this work.^42^^,^^43^

#### 2.1.1 Illumination channel

Visible illumination was provided by a white light LED (T7358; Prizmatix), filtered with a short and longpass filter (AT465lp; Chroma and FESH0650; Thorlabs) before being coupled into one arm of a bifurcated fiber (19 Fibre Y bundle, BF19Y2HS02; Thorlabs) using a custom LED-to-SMA coupler (Prizmatix). A laser diode (LP685-SF15 mounted in CLD1011LP; Thorlabs) was coupled into the second arm of the bifurcated fiber via a FC-PC single-mode patch cable (P1-630A-FC-1; Thorlabs) and an adjustable collimator (CFC-5X-B mounted in x-y-z adjustable mount CXYZ1/M; Thorlabs). An objective lens (40×/0.65 Plan Achromat Objective, RMS40X; Olympus) was used to couple light from the output port of the bifurcated fiber into the illumination channel of the PolyScope^®^ (PD-PS-0095, PolyScope^®^; PolyDiagnost). The bifurcated fiber, objective lens, and illumination channel of the PolyScope^®^ were cage mounted to allow for efficient coupling of light into the illumination channel of the PolyScope^®^. A custom 3-D printed mount was made to insert the illumination channel of the PolyScope^®^ into an adapter and cage mountable plate (AD16F and CXY1; Thorlabs). The illumination power at the distal end of the endoscope was measured with a thermal power meter (A-02-D12-BBF-USB, LaserPoint) and yielded 1.88±0.01  mW for the laser line and 0.52±0.01  mW for the broadband LED, well below the typically illumination power of a standard endoscope.

#### 2.1.2 Imaging channel

The image relayed by the PolyScope^®^ imaging fiber bundle was detected by two SRDAs [Fig. 1(c)]. The visible and NIR SRDAs (VIS: CMV2K-SSM4x4-9.2.10.3, NIR: SSM5x5 5.4.20.8, Imec; Belgium) consist of spectral filters monolithically deposited on a pixel level in a 4×4 and 5×5 grid pattern yielding 16 and 25 spectral bands, respectively, arranged in spectral macropixels [Fig. 1(a)]. Spectral data are thus acquired at the expense of spatial resolution. During the lithography process, spectral filters are deposited directly on the CMOS sensors of compact USB-3 xiQ cameras from Ximea. Each of these cameras weighs less than 30 g, is capable of recording video (90 fps), and performs still image acquisition with integration times ranging from 7.4  μs to 1 s.^44^^,^^45^ In this work, integration times ranged between 16 to 980 ms. The SRDAs used here cost >10000  USD each, but have potential to be manufactured for lower cost at high volume since the spectral filters are monolithically integrated on sensor during the lithography process.^45^

An infinity-corrected objective lens (Plan Fluorite 20×, UPLFLN20X; Olympus) was used to magnify the image of the fiber bundle, resulting in images of the fiber bundle of ∼700 and 1000 pixels diameter on the visible and NIR SRDA, respectively. Differences in the image size most likely arise from different distances between the objective and tube lens (the infinity space) in the two imaging channels but may also indicate slight misalignment in the optical components. A dichroic beamsplitter (652-nm single-edge dichroic, FF652-Di01; Semrock) was used to direct reflectance and fluorescence light toward a visible and NIR SRDA, respectively. A notch filter (685-nm laser notch filter, ZET685NF; Chroma) was placed in the fluorescence beam path to prevent laser light from saturating the NIR SRDA. Two 100-mm focal length tube lenses (Air-Spaced Achromatic Doublet, AR Coating 350-700, ACA254-100-A, and 650-1050 nm, ACA254-100-B; Thorlabs) were used to image the fiber bundle onto the SRDAs. A corner-mounted broadband mirror (21015; Chroma) was included in the reflectance channel for ease of alignment and to increase system compactness. The fluorescence and reflectance channels of the endoscope were separately aligned using an external light source (OSL2 with OSL2BIR bulb; Thorlabs) and the dichroic beamsplitter removed during alignment of the fluorescence imaging channel. The entire system was mounted in a black box (TB5 Black Posterboard, XE25L225/M, XE25L375/M Construction Rails and XE25W3 Quick Corner Cube; Thorlabs) on an optical breadboard (MB4545/M; Thorlabs) for ease of transport, yielding a system of 45×60×25  cm dimension. Each multispectral detector, typically the bulkiest element, measures only 26.4×26.4×24.6  mm and weighs less than 30 g.

### 2.2 Spectral Transmission Model

**Fig. 2:**
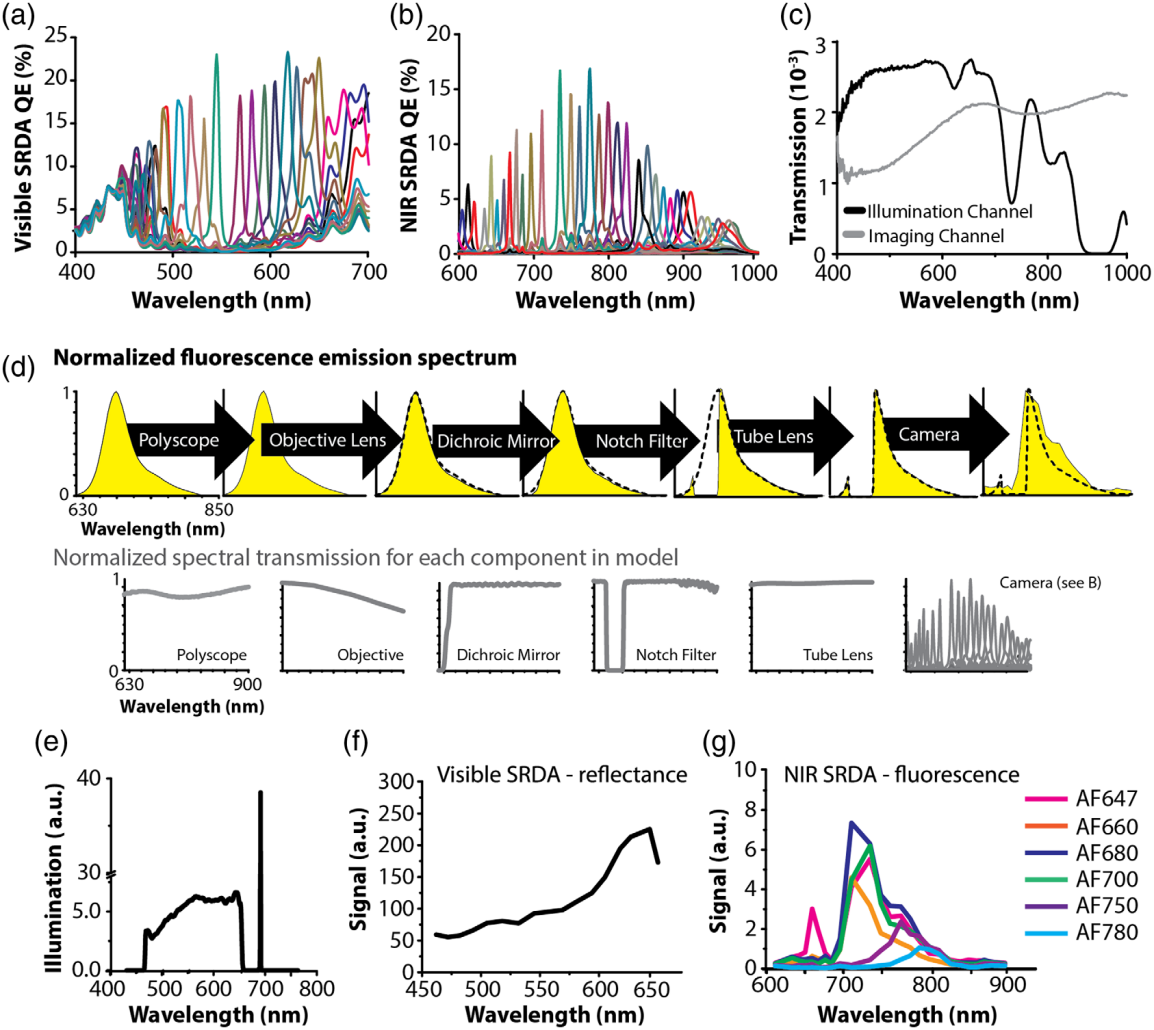
Overview of the spectral transmission model. (a) Quantum efficiency of the visible SRDA. (b) Quantum efficiency of the NIR SRDA. (c) Measured transmission of the PolyScope^®^ imaging and illumination channels, normalized to the area-under-curve. (d) Illustration of the fluorescence spectral channel propagation using AF660. The emission spectrum of the dye adjusted according to the spectral response of each component as it was propagated through the imaging channel. The spectrum at each stage is shaded in yellow and spectrum output from the previous component is shown in a black dashed line. (e) Modeled illumination provided by the white light LED and the laser line on sample. (f) Predicted spectrum of the reflected light from a uniformly reflecting target detected at the visible SRDA. (g) Predicted relative fluorescence signal for a range of dyes detected at the NIR SRDA [wavelengths shown in (f) and (g) represent the peak wavelength of the sampling spectral bands on the SRDAs].

To assess the influence of the spectral transmission characteristics of the system optics on the spectra that would be recorded by the SRDAs, we created a simple model (MATLAB^®^ 2015, Mathworks) to evaluate the potential spectral distortions introduced by the entire optical system, and in particular, the visible snapshot SRDA [Fig. 2(a)], the NIR SRDA [Fig. 2(b)], and the PolyScope^®^ [Fig. 2(c)]. The model was based on a previously published method of modeling the spectral transmission characteristics by linear multiplication of the normalized transmission spectra of the individual system components [Fig. 2(d)].^41^ The transmission spectra of the individual components were either obtained directly from the suppliers or experimentally measured using a stabilized halogen light source (SLS201; Thorlabs) and a spectrometer (AvaSpec-ULS2048-USB2-FCDC; Avantes). The sample illumination was modeled by propagating the spectra of the white light LED and the laser line through the endoscope’s illumination channel [Fig. 2(e)]. The predicted reflectance spectrum of the illumination only that would be detected by the visible SRDA is shown in Fig. 2(f). The purpose of the model was twofold as described below: to determine whether several fluorescent dyes could be accurately resolved by the system and to provide more accurate endmembers, which are the known spectral components input to the spectral unmixing algorithm.

#### 2.2.1 Analysis of fluorescence multiplexing capability

To predict the relative fluorescence signal strength produced from a fluorescent dye placed in the sample plane, the quantum efficiency (${\mathrm{QE}}_{\text{dye}}$), the extinction coefficient ($\varepsilon^{+}$), and the spectral overlap between the endoscopic illumination and the excitation spectra of several dyes were considered. The predicted fluorescence signal from six fluorescent dyes (AF647, AF660, AF680, AF700, AF750, and AF780; Invitrogen) was assessed using both published reference spectra (Invitrogen) and measured reference spectra of the dyes dissolved in phosphate-buffered saline (PBS, 10010015; Thermo Fisher), recorded on a plate reader (CLARIOstar; BMG LABTECH).

Relative fluorescence emissions were predicted for each of the dyes [$I_{\mathrm{em}}(\lambda )$] using

$$I_{\mathrm{em}}(\lambda )=I_{\mathrm{em}}^{\mathrm{norm}}(\lambda )\varepsilon^{+}{\mathrm{QE}}_{\text{dye}}\int I_{\mathrm{sample}}^{\mathrm{norm}}(\lambda^{\prime })I_{\mathrm{ex}}^{\mathrm{norm}}(\lambda^{\prime })d\lambda^{\prime },$$ (1)

where $I_{\text{sample}}^{\mathrm{norm}}(\lambda )$ is the predicted sample illumination spectrum, and $I_{\mathrm{ex}}^{\mathrm{norm}}(\lambda )$ and $I_{\mathrm{em}}^{\mathrm{norm}}(\lambda )$ are the excitation and emission spectra of each fluorescent dye (all normalized to the area under the curve). ${\mathrm{QE}}_{\text{dye}}$, $\varepsilon^{+}$, and the dye excitation and emission spectra were obtained from the supplier. Fluorescence signal was assumed to be linearly proportional to the dye extinction coefficient, the QE, and the light available for fluorescence excitation [$I_{\text{sample}}^{\mathrm{norm}}(\lambda )$]. A prediction of the fluorescence emission spectra that would be recorded by the NIR SRDA was made by propagating $I_{\mathrm{em}}(\lambda )$ through the combined spectral response of the imaging channel [Fig. 2(g)]. This allowed us to identify three fluorescent dyes for experimental evaluation that were likely to be resolved at high accuracy using the NIR SRDA (AF647, AF660, and AF700).

#### 2.2.2 Modeling endmembers for spectral unmixing

To derive useful information from the spectral data cube, it is necessary to relate the spectral data to the chemical composition of the sample. Such relationships can be established via spectral unmixing. When performing MSI, quantification is often achieved using linear spectral unmixing methods^40^ to resolve the relative contribution of different spectral components to the measured signal. Standard linear spectral unmixing methods require that spectra of components contributing to the signal be known a priori and input into the spectral unmixing algorithm. Independently measured reference spectra are often used for this purpose, however, this needs to be adjusted to account for the spectral transmission characteristics of a given system. Measured reference spectra for all chemical components under investigation in our experimental studies were propagated through the model to generate these adjusted endmembers. The calculation was repeated for illumination of a uniformly reflecting target to predict the spectrum of pure reflectance light that would be sampled by the visible and NIR SRDAs.

#### 2.2.3 Experimental evaluation of model accuracy

The transmission spectrum of the complete endoscope illumination channel was measured by coupling a stabilized broadband light source (SLS201; Thorlabs) to the input port of the bifurcated fiber in place of the white light LED. The broadband light transmitted to the distal end of the PolyScope^®^ was measured with a spectrometer (AvaSpec-ULS2048-USB2-FCDC; Avantes). The spectral transmission characteristics of the illumination channel of the endoscope were extracted by dividing the measured spectrum by the known spectrum of the stabilized broadband light source and the root-mean-square-error (RMSE) between the normalized modeled and experimentally measured transmission spectra was calculated.

The transmission spectrum of the complete endoscope imaging channel, including SRDAs, was measured by illuminating the distal tip of the endoscope via an optical fiber (M71L01; Thorlabs). Broadband light (OSL2 with the OLSB2 bulb for the reflectance channel and the OSL2BIR bulb for the fluorescence channel; Thorlabs) was passed through a monochromator (CM110 1/8m; Spectral Products) and wavelength scans between 400 and 900 nm in 3-nm increments were performed using LabVIEW^®^ to control the monochromator and cameras. The integration times of the visible and NIR SRDAs were set to 16 and 60 ms, respectively. The gain level of both cameras was set to 0.0 and 10 image frames were acquired at each wavelength increment. Before and after each scan, 20 dark frames with matched integration times and gain were captured to allow for dark subtraction of the acquired data.

The acquired data were averaged, dark subtracted, and converted to spectral response curves, as described previously.^40^ The response of the SRDA, as measured in digital numbers [DN(λ)], was taken as the average DN of a region of interest (RoI) drawn over the endoscope field-of-view (FoV). These experimentally measured spectral response curves for each SRDA arm were normalized to the highest peak intensity recorded in the reflectance and fluorescence imaging channel, respectively, and compared to the spectral response predicted by the model. The RMSE is reported as the mean±standard deviation across all spectral bands in each imaging arm to evaluate the accuracy of the model.

To evaluate the accuracy of the modeled endmembers, 40  μM solutions of the three fluorescent dyes (AF647, AF660, and AF700) in PBS were endoscopically imaged. Thirty microliters of each dye solution was placed in separate wells of a microwell plate (18 well, microslide, 81826; ibidi). The well plate was placed 10 mm under the distal tip of the endoscope. Two replicates of each dye solution were imaged along with two control wells containing 30   μL of PBS alone. The endoscopically measured reference spectra were extracted from 60 pixel radii circular RoIs placed over the wells of the well plate located using data acquired in the reflectance channel of the endoscope. Reference emission spectra of each of the three dyes, prepared at 60  μM concentration, were acquired with a plate reader (CLARIOstar; BMG LABTECH) and used to model endmember spectra. The RMSE of the modeled fluorescence spectra was subsequently evaluated by comparison with the experimentally acquired RoI spectra.

### 2.3 Multispectral Image Acquisition and Data Processing

For all imaging studies, data acquisition was performed in LabVIEW^®^ (National Instruments) and data analysis in MATLAB^®^ (The Mathworks).

Prior to image acquisition, 10 dark frames were captured to dark subtract the acquired image data. For each image acquisition of stationary targets, 10×10 bit image frames were simultaneously acquired with the visible and NIR SRDAs. The integration time of the visible and NIR SRDAs ranged between 30 and 980 ms, optimized to the imaging application according to the intensity of the sample illumination, the reflectance of the scene, and the endoscope working distance (WD). Averaged and dark subtracted images were demosaicked via linear interpolation between pixels in adjacent spectral macropixels to extract 16 and 25 spectral band MSI data cubes from the visible and NIR SRDAs, respectively.

**Fig. 3:**
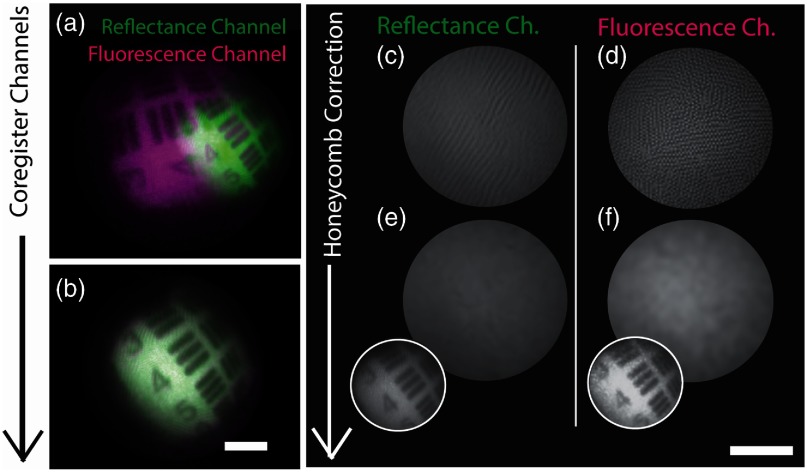
Coregistration of SRDA images. A test target containing resolution elements in group 1 of an USAF target were captured by the visible and NIR SRDAs in the reflectance and fluorescence channels (a) before and (b) after coregistration. The coregistered images were then comb-corrected to remove the pattern introduced by the individual fibrelets in the imaging fibre bundle of the PolyScope^®^. Images acquired from a uniformly reflecting target by the (c) visible and (d) NIR SRDAs were used to identify the position of the fiberlets. Comb-corrected images (e,f) were then produced via interpolation as described in the methods (scale bars=5  mm). The insets in panels (e) and (f) show the comb-corrected images of the USAF target. Note that the comb structure in panel (c) is slightly smeared in comparison to (d); this is due to slight misalignment of the mirror in the reflectance channel of the endoscope.

Due to the different beam paths in the reflectance and fluorescence imaging channels, the images recorded by the visible and NIR SRDAs were not naturally coregistered [Fig. 3(a)]. The MSI data cubes were coregistered by an affine transformation of the NIR MSI data cube [Fig. 3(b)] using the MATLAB^®^ fitgeotrans function. Corresponding coordinate points in the visible and NIR imaging channels were manually defined based on endoscopic images of a text target with strong salient features, externally illuminated with a broadband light source (OSL2 with OSL2BIR bulb; Thorlabs) using the MATLAB^®^ cpselect function. Coregistering via spatial features, rather than via the honeycomb pattern of the fiber bundle, was found more robust due to occasional misidentification of the fiberlets in the honeycomb pattern by the comb-correction algorithm.

After coregistration, comb-correction was performed [Figs. 3(c)–3(f)] according to previously published methods^46^ using images of a uniformly reflecting target (Sphere Optics Lambertian White Screen; SG3151-0) illuminated with a broadband light source (OSL2 with OSL2BIR bulb; Thorlabs) to visualize the pattern in both channels. To compensate for any misidentification of fiberlet centers, a Gaussian filter with standard deviation set to half the spectral macropixel dimensions (i.e., 2 pixels for the visible and 2.5 for the NIR SRDA) was applied to the individual MSI data cube images after comb-correction.

Finally, spectra within the MSI data cubes were max-min normalized to remove any systematic offsets and non-negativity least squares (LS) spectral unmixing was applied using the normalized reference endmembers output from the spectral transmission model. Pixels with a coefficient of determination below 0.8 were excluded from the resulting abundance maps. Provided the coregistration matrix has been previously defined, the full-image processing (including spectral unmixing) takes ∼10  min.

### 2.4 Technical Characterization of the Multispectral Endoscope

#### 2.4.1 Spatial resolution

The endoscope spatial resolution (limited by the imaging fiber bundle) was determined at two WDs by imaging a 1951 United States Air Force (USAF) test chart target (#53-714; Edmund Optics) with the visible SRDA only. The spatial resolution was determined by the Michelson contrast from individual spectral band images from the visible MSI data cube according to

$$C_{m}=\frac{Y_{\max}-Y_{\min}}{Y_{\max}+Y_{\min}}.$$ (2)

This was obtained by fitting a sinusoidal curve over the line pattern of a resolution element of the 1951 USAF target and extracting the maximum ($Y_{\max}$) and minimum ($Y_{\min}$) intensity from the fitted sine curve. Endoscopic illumination nonuniformities, common to endoscopic imaging^6^ arising from effects, such as vignetting, required that a polynomial baseline correction of the cross-sectional intensity profile was applied prior to extracting the Michelson contrast. Images were acquired using the internal illumination of the endoscope at WDs of 5 mm (camera integration time=480  ms and gain=0) and 10 mm (camera integration time=900  ms and gain=0).

#### 2.4.2 Barrel distortion and field-of-view

To characterize barrel distortion in the acquired images, a checkerboard of 1 mm square pattern was imaged at WDs between 2.5 and 15 mm in 2.5-mm increments. The checkerboard pattern was illuminated with an external light source (OSL2 with OSL2 bulb; Thorlabs) and three images from the MSI data cube of the visible SRDA (peak wavelength=462, 566, and 638 nm) were used for the evaluation using previously published methods.^42^ We identified the position of the center of the fiber bundle and plotted the radial distance to each vertex in the checkerboard pattern $r_{d}$ (in pixels) against the true distance $r_{u}$ (in mm) known from the dimensions of the checkerboard pattern.

When imaging a flat target in air, the effective FoV of the PolyScope^®^ is limited by the extent of the illumination rather than the optics of the imaging channel. The angular extent of the illumination cone was defined as the area within which the diffuse reflectance in all spectral bands in the visible SRDA exceeded 10% of the maximum reflected intensity. The diffuse reflectance was determined by acquiring images of a laminated white paper at WDs incremented from 2.5 to 15 mm in 2.5-mm increments.

To determine the angular FoV of the imaging channel, the protocol was repeated but with external illumination (OSL2 broadband light source with OSL2B bulb; Thorlabs). The imaging FoV of the endoscope was defined as the area within which the pixel values were larger than zero across all spectral bands in the visible SRDA. To more closely mimic endoscopic operation in the GI tract, barrel distortion and FoV were also measured under the following conditions: with water droplet added to the distal tip and with both the distal tip and imaging target fully submerged in water. The pixel radii of the circular FoVs were converted to mm, accounting for the barrel distortion at each WD. The angular FoV of the endoscope was extracted from the tangent of the gradient of the linear fit of $r_{\mathrm{FoV}}$ plotted against WD. Errors were calculated by addition of the fit standard error (one-sigma confidence interval) in quadrature. The camera integration times varied between 30 ms and 1 s according to the WDs and scenes imaged.

After these characterization steps, all subsequent imaging was performed at a 10 mm WD, as this gave a sufficient FoV and spatial resolution for our initial imaging performance evaluation of the spectral endoscope.

#### 2.4.3 Spectral fidelity

To characterize the uniformity of the spectral response over the endoscopic FoV, we defined a “spectral fidelity metric,” which expresses the MSE of a normalized pixel spectrum compared to that of the average spectral response across the FoV when imaging a uniformly reflective target. The spectral fidelity in the visible SRDA was determined by imaging (camera integration time=995  ms and gain=0) a uniformly reflective target (Lambertian White Screen, SG3151-0; Sphere Optics).

### 2.5 Oxygenation Phantom Imaging

#### 2.5.1 Phantom preparation and imaging

To evaluate the spectral imaging performance of the endoscope, we developed an agarose phantom containing two capillaries of chemically oxygenated and deoxygenated mouse blood and fluorescent dye inclusions of AF647, AF660, and AF700 dissolved in PBS. The phantom was designed such that the blood capillaries and the fluorescent dyes could be visualized within the same endoscopic FoV at a 10-mm endoscopic WD. The visible camera integration time was set to 980 ms and its gain to 0, while the NIR camera integration time was 980 ms with gain 5.

The phantom base material was prepared according to a previously published recipe^40^ and poured into a 120-mm diameter glass petri dish to form a 5-mm thick agarose layer. Four holes were made in the agarose slab using a transparent straw with 3-mm internal diameter (391SIPCL; Plastico). Four 5-mm long pieces of transparent straw, of which one side was sealed with a glue gun (PA6-GF30; Type PX 06; Henkel Pattex Supermatic), were placed in the holes with the glue-sealed end facing down. Each straw was filled with a 80-μM pure fluorescent dye solution of AF647, AF660, or AF700 dissolved in PBS and one hole filled with a PBS control.

Mouse blood was obtained from seven female C3H/HeOuJ mice (6 to 10 weeks old) after euthanasia by exposure to raising ${\mathrm{CO}}_{2}$ concentration. One minute after the complete cessation of respiratory movements, about 0.5 mL of blood was collected by cardiac puncture from each mouse and placed in a microtube containing 10  μL of heparin sodium salt. Animal handling and euthanasia complied with the British schedule 1 of the Animals (Scientific Procedures) Act 1986. The mouse blood was chemically deoxygenated following a protocol developed by Briley-Sæbø and Bjørnerud.^47^ Deoxygenation was achieved by adding, and thoroughly mixing, 1.5 mg of sodium dithionite (157953-5G-D; Sigma Aldrich) with 0.5 mL of heparinized blood. Successful deoxygenation of the blood was verified with a dissolved oxygen monitor (OxyLite with NX/BF/OT/E probe; Oxford Optronix) on which the deoxygenated blood had a partial oxygen pressure below the detection range of the sensor. Blood was chemically oxygenated by adding 1  μL of 30% hydrogen peroxide (216763-100ML; Sigma-Aldrich) to 0.5 mL of blood. The partial oxygen pressure in the oxygenated blood saturated the dissolved oxygen monitor, indicating a partial pressure higher than 200 mmHg. Deoxygenated and oxygenated blood were subsequently pulled into separate 100-mm glass capillaries (CV1518; CM Scientific) via capillary action. The capillaries were sealed using capillary tube sealant (02678 Fisherbrand Hemato-Seal Capillary Tube Sealant; Fisher Scientific). Reference spectra of the blood capillaries and the phantom base material were acquired with a calibrated bifurcated reflection probe (RP29, Reflection Probe with Linear Leg; Thorlabs) and spectrometer (AvaSpec-ULS2048-USB2-FCDC; Avantes). Successful oxygenation and deoxygenation was further verified by qualitative comparison of these spectra with tabulated literature data.^48^ A reference spectrum of the endoscope white light LED source was obtained by placing the reflection probe flush against the reflectance target. The phantom was imaged three times using the multispectral endoscope, repositioning the phantom between each repeat.

#### 2.5.2 Phantom data analysis

**Fig. 4:**
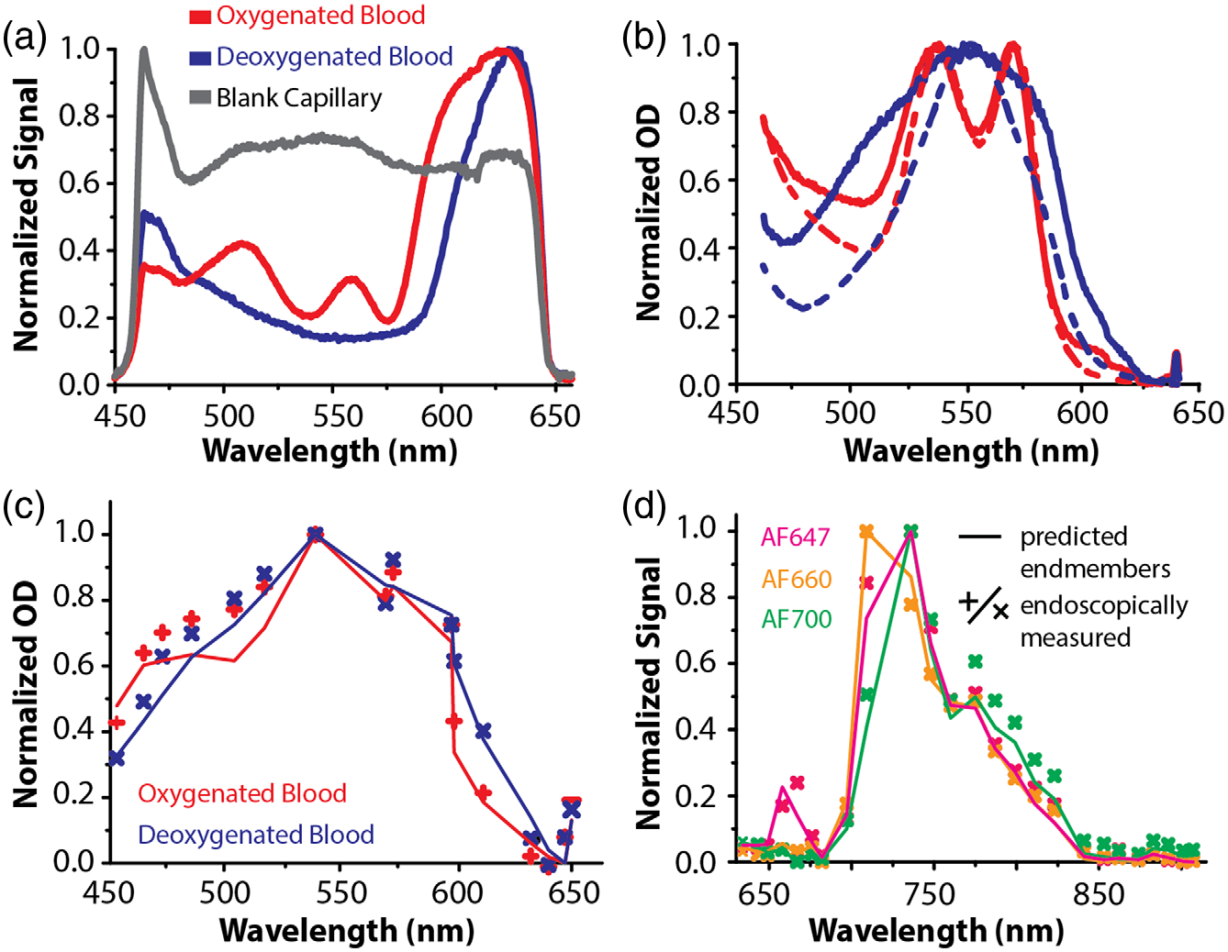
Calculation of endmembers for spectral unmixing of reflectance and fluorescence imaging data using the spectral transmission model. (a) Reference reflectance spectra measured from glass capillaries containing oxygenated and deoxygenated blood, and a “blank capillary” containing only PBS. (b) The corresponding optical density (OD) spectra calculated from the experimentally measured reference spectra shown in panel (a) compared to literature values (dashed lines).^48^ (c) Comparison of predicted endmembers (solid line) and the endoscopically measured spectra of the oxygenated and deoxygenated blood (points). (d) Comparison of predicted endmembers (solid line) and the endoscopically measured spectra of the fluorescent dyes (points). Max–min normalization was performed on the predicted endmembers and the endoscopically measured spectra.

Endmember spectra for the fluorescent dyes were obtained by propagating the reference fluorescence spectra, previously acquired on the plate-reader, through the spectral transmission model. Endmember spectra for spectral unmixing of the oxygenation status of the blood were obtained by propagating the acquired reference reflectance spectra [Fig. 4(a)] through the spectral transmission model. The propagated reflectance spectra of the blood capillaries ($I_{\text{blood}}$) and Lambertian white screen ($I_{\text{white}}$) were used to calculate optical density (OD) spectra:

$$\mathrm{OD}=\log(\frac{I_{\text{white}}}{I_{\text{blood}}}).$$ (3)

The log of the spectral band images acquired in the reflectance channel of the endoscope, divided by the spectral band images of the reflectance target, was calculated to obtain the OD of the MSI reflectance data cube prior to spectral unmixing.

### 2.6 Multiplexed Fluorescence Imaging in a Pig Esophagus

Endoscopic imaging was performed in an *ex vivo* porcine model to more closely mimic the real clinical imaging conditions of multiplexed fluorescence imaging within a hollow lumen with variable WD. An air-inflated pig stomach and esophagus (Medical Meat Supplies) was used as an endoscopic imaging phantom. All blood had been drained from the pig stomach and esophagus.

Agarose dye plugs were prepared using a warm 6.0% agar solution (05039-500G; Fluka) mixed with equal parts of 160  μM fluorescent dye solution (AF647, AF660, and AF700) in PBS. Before the solution cooled and solidified, droplets of the solution were pipetted onto a glass slide and formed ∼5-mm diameter and 1- to 3-mm thick plugs after solidification. The dye plugs naturally adhered to the internal esophagus surface due to stickiness and high surface tension. They were placed inside the pig esophagus using biopsy forceps threaded through the accessory channel of a clinical gastroscope (GIF-1T240; Olympus). The AF647, AF660, and AF700 dye plugs were separately placed into the esophagus. Therefore, the multispectral endoscope, operated by a trained endoscopist (Med. Dr. Wladyslaw Januszewicz), was threaded through the accessory channel of the clinical gastroscope, and MSI data were continuously acquired during an endoscopic withdrawal. The integration time of both the visible and NIR SRDA was set to 990 ms and the gain to 0. Image averaging was not possible with such a long integration time since the endoscope moved during data acquisition inside the esophagus. During data acquisition with the multispectral endoscope, the internal illumination source of the clinical gastroscope was switched off.

After imaging, the pig esophagus was sliced open and the dye plugs placed directly on the opened organ. Reference fluorescence spectra of the dye plugs and the tissue background were acquired with the calibrated spectrometer and an inspection fiber (M71L01; Thorlabs and AvaSpec-ULS2048-USB2-FCDC; Avantes) while illuminated by the multispectral endoscope. The acquired reference spectra were smoothed with a Savitzky–Golay filter (order=3, frame length=17) and propagated through the spectral transmission model to calculate endmembers.

## 3 Results

### 3.1 Accuracy of the Spectral Transmission Model of the Endoscope

The spectral transmission model served a dual purpose: to optimize the selection of optical components for multiplexed detection of several fluorescent dyes and to provide endmembers for spectral unmixing. The accuracy of the model was determined by comparing the predicted spectral transmission characteristics of the illumination and imaging channels to those measured experimentally. A RMSE of 0.003 was obtained between the modeled and experimentally measured transmission spectra of the illumination channel of the endoscope. Similarly, low RMSE was obtained when comparing the modeled and experimentally measured spectral response of the imaging channel in the reflectance (RMSE: 0.08±0.02) and fluorescence (RMSE: 0.04±0.02) arms, where the error is cited as the standard deviation across the spectral bands of the SRDAs. The low RMSEs indicate high agreement between the model and the experimental data, indicating that the simple multiplicative model had predictive power. Based on the experimental characterization data in the 400 to 900 nm range, taking into account the spectral response of the imaging channels and the filters of the SRDAs, the peak wavelengths of the spectral response in the reflectance imaging channel lie in the range from 463 to 648 nm with FWHMs ranging between 9 and 26 nm. For the fluorescence channel, the peak wavelengths ranged between 659 and 891 nm, and the FWHM between 7 and 15 nm.

Endmembers were modeled by propagating reference reflectance and fluorescence spectra through the respective channels. For reflectance imaging, reference spectra were measured from sealed capillaries containing fully oxygenated or deoxygenated blood using a spectrometer [Fig. 4(a)]. To compare these measured spectra to the literature, the values were converted into optical density and then normalized [Fig. 4(b)]. Similar trends in spectral shape could be observed. Discrepancies between reference and measured blood spectra may arise from the glass capillary in which the blood was contained, the chemicals used to oxygenate or deoxygenate the blood and possibly also from slight variations in the illumination used during acquisition. The reflectance endmembers for oxygenated and deoxygenated blood were then calculated using the model and compared to spectra obtained directly using the imaging channel of the endoscope by extracting the average spectra from RoIs placed over the capillaries [Fig. 4(c)]. RMSEs of 0.12 and 0.10 were found when comparing the predicted and experimental results. The predicted and measured RoI fluorescence spectra for three fluorescent dyes were also in good agreement [Fig. 4(d); RMSE: 0.03±.01].

### 3.2 Technical Characterization of the Endoscope Performance

#### 3.2.1 Angular field-of-view

**Fig. 5:**
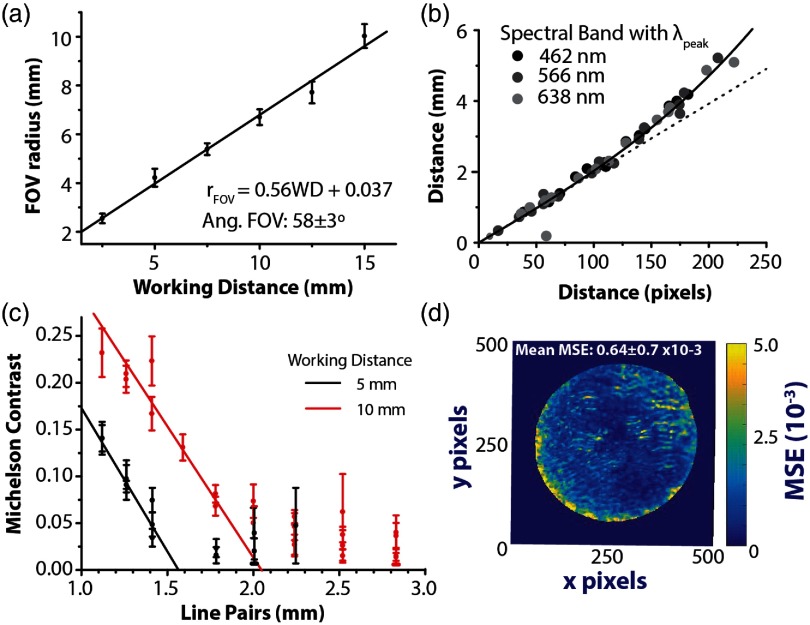
Technical characterization of the endoscope performance. (a) The FoV of the endoscope when operating in air (as defined as the angular extent of its illumination cone) was measured at a range of WDs, from which an angular FoV of 58±3  deg was determined. (b) To extract the radius of the FoV at a specific WD, a checkerboard pattern was first imaged to characterize the barrel distortion. The barrel distortion was extracted by fitting the true radial distance from the centre of the FoV to each vertex in the checkerboard pattern $r_{d}$ (in pixels) against the true distance $r_{u}$ (in mm). (c) The spatial resolution of the endoscope at WDs of 5 and 10 mm was determined by imaging a USAF test chart target and plotting the Michelson contrast against the number of line pairs/mm. (d) “Spectral fidelity” was calculated spatially to assess the uniformity of the spectral response across the endoscopic FoV. The spectral fidelity map shows the MSE of the normalized spectrum in each pixel compared to the FoV average.

The FoVs defined by the extent of the light cone emerging from the illumination channel of the endoscope were measured to be 58±3  deg, 65±4  deg, and 52±1  deg when operating: in air [Fig. 5(a)], with a droplet on tip, and fully submerged in water, respectively. The respective FoVs defined by the imaging channel (measured using external illumination) were 102±8  deg, 96±5  deg, and 84±5  deg. As expected, in both cases, the FoV is reduced when the endoscope operates immersed in water, due to the change in refractive index. Within experimental uncertainties, the FoV of the imaging channel when operating in air agrees with the supplier specified FoV of 120±10  deg.^49^ Differences between imaging of a flat target and within a tubular structure (according to the intended use) may explain the discrepancies between the FoV of the illumination and imaging channel,^49^ which were here measured using a flat target.

#### 3.2.2 Barrel distortion

For each WD, the barrel distortion of the endoscope was characterized by imaging a checkerboard pattern and plotting the radial distance to each vertex in pixels ($r_{d}$) against the true distance ($r_{u}$) in mm, for three spectral bands in the visible SRDA [Fig. 5(b)]. In line with expectation, no distinctive wavelength dependence could be observed and data from the three spectral bands were therefore grouped in the analysis. The radii of the FoVs of view measured in pixels were converted into mm, taking the effect of the Barrel distortion into account. Due to the limited Barrel distortion observed, no barrel distortion correction was performed during image processing.

#### 3.2.3 Spatial resolution

The Michelson contrast for a set of resolution elements of 1951 USAF test chart target was evaluated at endoscopic WD of 5 and 10 mm to determine the spatial resolution of the endoscope [Fig. 5(c)]. A linear fit was applied to the data and the spatial resolution was extracted based on a Michelson cut-off contrast of 10%. The smallest resolvable line pairs/mm were 1.7±0.6 and 1.3±0.2 at 5 and 10 mm WD, respectively; this corresponds to spatial resolutions of 300±100  μm and 400±100  μm. The uncertainty was calculated by adding the standard errors of the fit parameters (one sigma confidence interval) in quadrature.

#### 3.2.4 Spectral fidelity

The uniformity of the spectral response across the endoscopic FoV was characterized by imaging a uniformly reflecting target. “Spectral fidelity,” evaluated spatially, is calculated as the mean-squared-error (MSE) of a given pixel spectrum compared to the average spectral response across the FoV. A high “spectral fidelity” (i.e., low average MSE of 0.0064±0.0070) was observed [Fig. 5(d)]. Some degradation in the spectral fidelity could be observed toward the edges of the endoscopic FoV, which may arise from slight chromatic aberrations or a decrease in the performance of the honeycomb correction toward the edges of the FoV. The nonsymmetric nature of the degradation further suggests a slight tilt in one of the optical components in the imaging path. Nonetheless, based on the small overall difference in the spatial MSE, we can assume that such effects are small and would not be expected to impact spectral unmixing.

### 3.3 Phantom Imaging Results

**Fig. 6:**
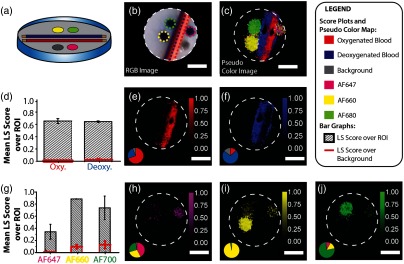
Validation of performance in an agarose phantom. (a) Schematic of the phantom. Chemically oxygenated and deoxygenated mouse blood was held in capillaries and positioned between fluorescent dye inclusions (AF647, AF660, and AF700) within the agarose phantom. (b) Standard RGB photo of the constructed phantom. After spectral unmixing, a pseudocolor map revealed the positions of fluorescent dye inclusions and the oxygenation status of the blood, which were not apparent from the RGB image alone (c). (d) Quantitative data extracted from the reflectance channel of the endoscope. The LS scores were extracted from RoIs placed over (e) oxygenated and (f) deoxygenated blood abundance maps obtained after spectral unmixing. The error bars indicate the range of LS score over repeat measurements; the LS score of the background is shown in red. (g) Quantitative data from the fluorescence channel were extracted from abundance maps of (h) AF647, (i) AF660, and (j) AF700 obtained after spectral unmixing (scale bars=5  mm). Pie charts denote overall LS scores in RoIs drawn in the abundance maps to illustrate crosstalk between endmembers.

An agarose phantom [Figs. 6(a) and 6(b)] was prepared to evaluate the potential of the endoscope for simultaneous spectral imaging of reflectance and multiplexed fluorescence. The reflectance imaging channel was dedicated to unmixing blood oxygenation status and the fluorescence channel to unmixing the signal from three fluorescent dyes (AF647, AF660, and AF700). After spectral unmixing using the modeled endmembers [Fig. 4(c)], a pseudocolor map revealed the oxygenation status of the blood and identified the fluorescent dyes [Fig. 6(c)]. The spectral unmixing performance within a RoI drawn over the location of the capillary or dye inclusion was demonstrated [Figs. 6(d)–6(j)] according to three criteria: the LS score of the expected endmember; the LS score of the background; and the degree of misfitting to the other endmembers. The blood capillaries [Figs. 6(d)–6(f)] and the fluorescent dye inclusions [Figs. 6(g)–6(j)] could each be identified from abundance maps after spectral unmixing, by taking a majority decision based on the LS scores of the different endmembers.

The SBR of a given endmember was then calculated as the ratio of the LS score assigned to the correct endmember within the RoI to that incorrectly assigned to the background (defined as the area outside the RoI). The SBR and spectral abundance of the blood capillaries and dyes in the phantom were oxygenated blood: SBR=94±22, abundance=69±5%; deoxygenated blood: SBR=43±33, abundance=77±5%; AF647: SBR=35±10, abundance=42±6%; AF660: SBR=9±2, abundance=95±3%; and AF700: SBR=6±2, abundance=83±2%). The SBR and abundance values are cited as the mean and the range across the three repeat image acquisitions.

### 3.4 Imaging of Fluorescent Dyes in an Ex Vivo Pig Esophagus

**Fig. 7:**
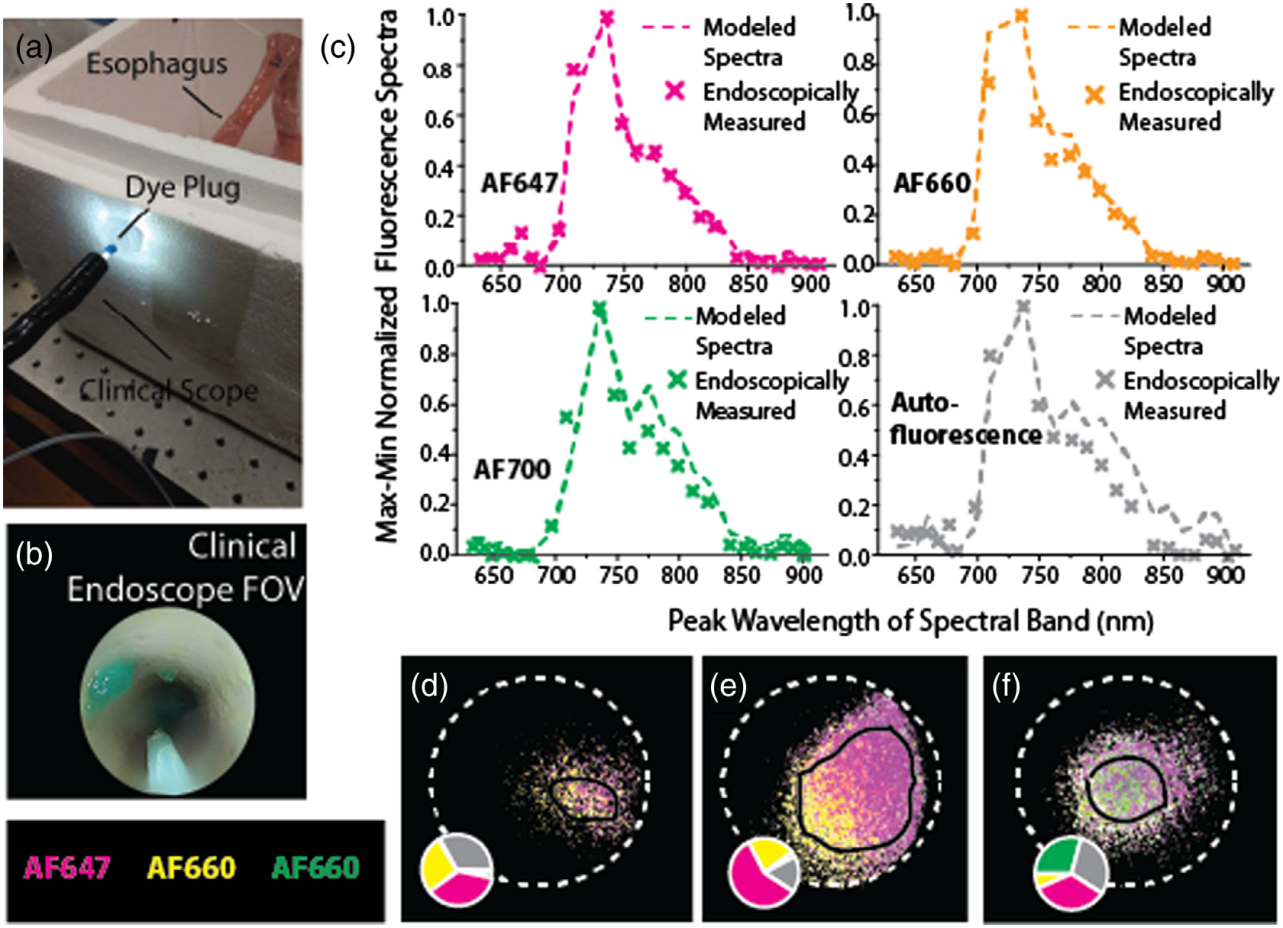
Endoscopic imaging in an insufflated pig esophagus. (a) Three fluorescent dye plugs were placed inside the esophagus. (b) An image of a fluorescent dye plug, internally placed in the pig esophagus, acquired with the clinical gastroscope, through which the spectral endoscope was inserted. (c) The endoscopically acquired MSI data were spectrally unmixed using endmembers modeled based on reference fluorescence spectra of the dye plugs and the tissue autofluorescence. (d–f) Pseudocolor maps of the unmixed spectral fluorescence signals from the three fluorescent dye plugs. The RoIs, from which their respective spectra and abundances (shown as pie charts) were extracted, are indicated by the black line and the FoV by a dashed line.

An insufflated pig stomach and esophagus [Fig. 7(a)], with three internally placed fluorescent dye containing agarose plugs [Fig. 7(b)], was used as an endoscopic imaging phantom to simulate endoscopic multiplexed fluorescence imaging. Images from the spectral endoscope were continuously acquired during an endoscopic withdrawal. As all blood had been drained from the pig stomach and esophagus, only fluorescence imaging data were analyzed.

The acquired data were spectrally unmixed using endmembers modeled based on reference fluorescence spectra acquired from the dye plugs and the pig esophagus tissue. The modeled endmembers compared favorably (RMSE: 0.05±0.03; mean±std across the three endoscopically imaged dyes and the tissue autofluorescence) to those measured directly in the endoscopic images [Fig. 7(c)], although some discrepancies could be observed between the spectra of AF700 and the tissue autofluorescence spectra.

According to expectation, three fluorescent dyes were identified in the spectrally unmixed image data from the endoscopic withdrawal. The dye plugs could not be identified in images from the visible SRDA due to limited contrast, which meant a fully quantitative analysis could not be performed as there were no reference images to guide RoI placement. Instead, RoIs were placed on the basis of the observed unmixed fluorescence signal relative to the clinical endoscope FoV (not coregistered) and the abundance of each endmember over the RoIs was calculated [Figs. 7(d)–7(f)]. Based on the abundance, the three fluorescent dyes could not be distinguished via majority decision, perhaps due to the less distinct spectral features observed from the dye plugs [Fig. 7(c)] as compared to the dyes when dissolved in PBS [Fig. 4(d)] or to the lower SNR from the inability to average multiple measurements due to motion and changing WD.

## 4 Discussion

We report here the design, technical characterization, and first imaging results of a prototype bimodal multispectral endoscope based on SRDAs. Our preliminary imaging study demonstrates the potential of the endoscope to simultaneously record both reflectance and fluorescence imaging data. Our technical characterization was performed mainly in air using flat imaging targets. Although this does not represent the realistic imaging environment of a lumen, it provides a baseline against which performance of different endoscopes can be easily compared. The technical performance was in line with that expected based on manufacturer specifications of the PolyScope^®^ and our previous studies.^42^ When imaging an agarose phantom, we were able to detect and correctly identify the location of oxygenated and deoxygenated blood, using data recorded by the visible SRDA, and three fluorescent dyes, using data recorded by the NIR SRDA. We then implemented the endoscope in a clinically realistic scenario by introducing it into the accessory channel of a commercial white light gastroscope and imaging an *ex vivo* pig esophagus. We introduced the same three fluorescent dyes into the esophagus; in this scenario, the endoscope could detect the presence of the dyes within the lumen, but the ability to identify each dye was limited. This may be due to the less distinctive spectral features of the dyes when incorporated into the agarose dye plugs.

In the process of endoscope design and assembly, we developed a spectral transmission model to predict the endmembers of different imaging components for accurate spectral unmixing. Despite simplistic modeling of coupling losses and fluorescence, the resultant model was shown to have predictive power. It may be possible to use such a model in future to predict the imaging performance of spectral endoscopes prior to system assembly, helping to select appropriate optical components. The model could also be further developed using ray tracing software to increase the accuracy with which coupling losses and chromatic aberrations are dealt with.

Despite showing some promising results with this initial prototype, further developments are needed to promote multispectral endoscopy based on SRDA technology toward clinical application. First, the current system is unable to detect fluorescent dyes at clinically relevant concentrations. The typical molarity of fluorescent contrast agents after binding to a specific molecular target in the GI tract is often cited as being <100  nM.^29^^,^^50^^,^^51^ Fluorescent dyes solutions used here were prepared at >40  μM concentration, at least 2 orders of magnitude higher. The discrepancy in sensitivity of our system may arise due to the low intensity of sample illumination; coupling losses in the imaging channel; and the low quantum efficiencies of the SRDAs used in the study, which are not optimized for low light applications. These limitations could be overcome in future by increasing the illumination intensity, which is currently around fivefold lower than commercial endoscopes;^42^ optimizing the optical components used in the imaging channel; optimizing integration times; and depositing customized spectral filters optimized to detection of the specific fluorescence emissions required on specialist sensors with high quantum efficiency in the NIR range. Appropriate optimization of the spectral filters deposited on-chip could also remove the need for the dual channel methodology used here, removing the losses associated with the use of the dichroic mirror and notch filter. Since SRDAs acquire data via an aperture division approach, optimization of the spectral filters could also allow imaging at a higher spatial resolution by reducing the number of filters.^52^^,^^53^

A second limitation is the use of complete chemical oxygenation and deoxygenation of the blood in the phantoms, which does not represent physiological blood oxygenation levels. Alternative blood flow phantoms that allow the study of physiologically relevant blood oxygenation levels should therefore be tested in the future. The phantoms could also be extended to further explore the tissue contrast accessible from other endogenous chromophores in the visible spectral region, such as endogenous fluorophores like NAD(P)H, flavins, collagen, and elastin.

The PolyScope^®^ platform would facilitate initial clinical testing of our endoscope device; however, real-time display of the spectrally unmixed data is currently lacking. The operating endoscopist would need access to these data at video-rate, ideally with automated exposure time control and integrated averaging to maximize image contrast and signal-to-noise ratio. This could be achieved by implementing the image preprocessing and spectral unmixing algorithms on a graphical processing unit, but was beyond the scope of the present prototype study. The long integration times required to achieve acceptable SNR in the fluorescence channel of the endoscope is the main factor limiting the frame rate. After implementation of the aforementioned system improvements to increase the detection sensitivity, data acquisition could be performed at the intrinsic frame rate of the cameras without sacrificing spectral resolution. Data processing and display would then become the factors limiting speed.

With the aforementioned developments in instrumentation and data analysis, the integration of SRDAs for multispectral endoscopy may provide significant cost and size advantages over conventional approaches. Currently, SRDAs are expensive, but do have potential to be produced at low cost at high-volume manufacture since spectral filters are monolithically integrated on sensor during the lithography process.^45^ In particular, since the spectral filters are deposited directly on sensor, it is also conceivable that SRDA technology could be miniaturized to a scale that would allow chip-on-tip multispectral video endoscopy, enabling tissue contrast accessible from spectral reflectance imaging to be combined with that from multiplexed OMI to improve the diagnostic performance of endoscopic cancer screening. With the further developments suggested already, the current system could be a useful clinical tool, having the ability to simultaneously perform wide-field imaging and acquire optical biopsies via the acquisition of spectral data.

The benefit of the SRDA approach to endoscopy is that data can be simultaneously acquired in all spectral bands simultaneously using a single sensor. As suggested above, an SRDA could therefore be integrated as a “chip-on-tip” at the distal end of a commercial endoscope in future. This is beneficial when compared to multispectral systems based on filter wheels^21^^,^^30^^,^^31^ and tunable filters,^23^^,^^32^ which sacrifice temporal resolution to sequentially acquire spectral data. Sequential acquisition of spectral data can also introduce motion artifacts between sequentially acquired spectral band images. For such systems, the coordinate transformation matrices between all sequentially adjacent wavelengths must be recalculated continually to correct for motion artifacts. By contrast, in the SRDA approach presented here, images within each spectral band are inherently coregistered. Therefore, in our case, only one transformation matrix (to coregister the reflectance and fluorescence channels) is required, and it is computed only once, after alignment. If employed in future in a “chip-on-tip” scenario using a bespoke SRDA with appropriate spectral bands, no such coregistration would be needed.

Clinically mature spectral endoscope systems already exist that simultaneously acquire spectral band images. These generally operate by spectrally separating the light and directing each component to a dedicated 2-D sensor.^10^^,^^27^^,^^29^ Since each spectral band is acquired using a different sensor, unlike in the present prototype, such beam splitting approaches can be easily optimized to enable fluorescence imaging at clinically relevant detection sensitivities, for example, using cooled sCMOS detectors. It should also be noted that highly compact, robust, and low-cost grating based HSI systems with high spectral resolution have previously been demonstrated.^38^^,^^54^ These systems tend to rely on spatial scanning of the sample, which in many cases requires spatiospectral coregistration and prevents the video-rate acquisition of spectral data critical to many clinical applications. While such systems may not be suitable for wide-field inspection of the full esophageal lumen, they can provide detailed spectral information during inspection of smaller suspicious lesions, when real-time data acquisition and display are not as critical. By comparison, SRDAs instead sacrifice spatial resolution to acquire multispectral data in real time, making an SRDA approach more suitable for wide-field real-time endoscopic inspection of the full esophageal lumen.

## 5 Conclusions

Extraction of 3-D data (x-y-λ) from typically 2-D sensors (x-y) often requires conversion of spectral data to either the spatial or temporal domain. Application-dependent trade-offs between spatial, temporal, and spectral resolution need to be made. This work demonstrates a bimodal multispectral endoscope for simultaneous spectral reflectance and multiplexed fluorescence imaging, achieved by integrating two SRDAs into a clinically approved endoscope. In the process of endoscopy design and assembly, we developed a spectral transmission model that was used to predict the endmembers needed for spectral unmixing. Concurrent imaging and unmixing of oxygenated and deoxygenated blood together with the signal from three fluorescent dyes were demonstrated in an agarose phantom and in an *ex vivo* pig esophagus. This work shows that SRDAs can be used to perform simultaneous multispectral reflectance and multiplexed fluorescence imaging in endoscopy with acceptable spatial and spectral resolution. An SRDA approach to spectral endoscopy also has the potential to meet the constraints on compactness, robustness, and cost-effectiveness required for widespread clinical implementation. With further developments, SRDA technology has potential to improve image contrast available during endoscopic screening of the GI tract.
